# Are Synapse-Like Structures a Possible Way for Crosstalk of Cancer with Its Microenvironment?

**DOI:** 10.3390/cancers12040806

**Published:** 2020-03-27

**Authors:** Irina V Alekseenko, Igor P Chernov, Sergei V Kostrov, Eugene D Sverdlov

**Affiliations:** 1 Shemyakin-Ovchinnikov Institute of Bioorganic Chemistry of the Russian Academy of Sciences, 117997 Moscow, Russia; irina.alekseenko@mail.ru (I.V.A.); igor_ch@ibch.ru (I.P.C.); 2 Institute of Molecular Genetics, Russian Academy of Sciences, 123182 Moscow, Russia; kostrov@img.ras.ru; 3 FSBI «National Medical Research Center for Obstetrics, Gynecology and Perinatology named after Academician V.I. Kulakov» Ministry of Healthcare of the Russian Federation, 117198 Moscow, Russia

**Keywords:** immunological synapse, tumor microenvironment, cancer, cancer-associated fibroblast, direct interaction, synapse like interactions

## Abstract

The failure of therapies directed at targets within cancer cells highlight the necessity for a paradigm change in cancer therapy. The attention of researchers has shifted towards the disruption of cancer cell interactions with the tumor microenvironment. A typical example of such a disruption is the immune checkpoint cancer therapy that disrupts interactions between the immune and the cancer cells. The interaction of cancer antigens with T cells occurs in the immunological synapses. This is characterized by several special features, i.e., the proximity of the immune cells and their target cells, strong intercellular adhesion, and secretion of signaling cytokines into the intercellular cleft. Earlier, we hypothesized that the cancer-associated fibroblasts interacting with cancer cells through a synapse-like adhesion might play an important role in cancer tumors. Studies of the interactions between cancer cells and cancer-associated fibroblasts showed that their clusterization on the membrane surface determined their strength and specificity. The hundreds of interacting pairs are involved in the binding that may indicate the formation of synapse-like structures. These interactions may be responsible for successful metastasis of cancer cells, and their identification and disruption may open new therapeutic possibilities.

## 1. Introduction.

### 1.1. The Necessity of Changing the Paradigm in Cancer Therapy

The Cancer Genome Atlas (TCGA) project revealed ~10 million mutations associated with cancer [1]. Nonetheless, this enormous number of mutations does not reflect the entirety of the complexity of cancer (for the definition of complexity, see Reference [2]). The study revealed that the heterogeneity among cancer cells was much higher than previously estimated [3]. Each human tumor was found to contain 4–8 heterogeneous clones. The presence of various clones and cells that differ in their genotype and/or phenotype is at the root of the underlying problem of inefficient cancer therapy, and this problem is magnified by epigenetic, metabolic, and other types of heterogeneities. Any therapy applied to a heterogeneous mixture of cancer cells will induce different responses in different cells and may be inefficient in eliminating specific clones. Changes in the intratumoral heterogeneity during tumor development predetermine failures of targeted cancer therapies directed at the individual molecular components of cancer cells [3,4].

However, the main problem is that cancer is a “complex system” [2,5] composed of interacting subunits. These interactions result in the appearance of emergent properties characteristic to the whole system [6,7,8,9], properties that cannot be predicted from the properties of the individual subunits [10]. In cancer, the intratumoral complexity of the true cancer cells [11,12,13,14,15] should be distinguished from the complexity. This is due to their interaction with the tumor microenvironment (TME) [16,17]. 

The main tumor complexity is probably due to a large number of interactions between the true (usually epithelial) cancer cells and various cells of the TME [16]. Therefore, it is not surprising that the vast resources spent in the era of molecular targeted therapy have yielded only a few relatively efficacious agents. These agents include imatinib for the treatment of myeloid leukemia, trastuzumab directed at the human epidermal growth factor receptor 2 (HER2) expressed in some patients with breast cancer, and vemurafenib for melanoma expressing a mutant BRAF gene [18,19,20]. This emphasized the necessity of changing the paradigm in cancer therapy, and consequently, the attention of researchers gradually shifted towards the disruption of cancer cell interactions with the TME.

### 1.2. A Brief Description of the TME and Its Importance for Cancer Progression

The American National Cancer Institute defines the TME as “The normal cells, molecules, and blood vessels that surround and feed a tumor cell.” A tumor can change its microenvironment, and the microenvironment can affect how a tumor grows and spreads. (https://www.cancer.gov/publications/dictionaries/cancer-terms/def/tumor-microenvironment). The components of the TME constitute a complex mixture of different cells and extracellular material. The cellular component includes cells of a mesenchymal origin, i.e., the fibroblasts, the cancer-associated fibroblasts (CAFs), the myofibroblasts, the mesenchymal stem cells, the adipocytes, and the endothelial cells. It also includes cells of the hematopoietic origin, namely, the lymphoid cells (the T, the B, and the NK cells) and the myeloid cells (macrophages, neutrophils, and the myeloid-derived suppressor cells) [21,22,23,24,25]. The non-cellular component is represented by the extracellular matrix [26,27,28]. Cancer and stromal components form an integrated and evolving system with multiple interactions and emergent properties [26,29,30,31,32]. In their evolution, all tumors use a wide repertoire of healthy cells and adapt them to their conditions. The recruited normal cells facilitate the acquisition of the tumor-specific traits and form an ecological tumor niche that plays a significant role both in the development of the primary tumor and its metastasis [26,27,33,34,35,36,37].

Due to the interaction of cancer and stromal cells, tumors evolve as organ-like entities. These interactions include (i) direct binary contacts between ligands and receptors exposed on the surface of cancer and stromal cells, and (ii) paracrine communication between cancer (usually epithelial) cells and various TME cells [38,39]. Some authors use the term “symbiotic” for tumor–stroma interactions [40,41]. Stromal cells modified by the malignant epithelium form a permissive microenvironment that controls the cancer progression [21]. The symbiosis of cancer and stromal cells includes a complimentary exchange of paracrine factors affecting the TME characteristics. The most important consequence of this exchange is the transformation of normal fibroblasts into cancer-associated fibroblasts (CAFs). 

It is important to note that due to diffusion, paracrine signals can be transmitted over distances of tens of cell diameters [38], forming a gradient of signals that, depending on the concentration, can induce different responses instead of a simple “yes” or “no” binary responses. The transmission of signals will presumably be efficient only between closely located cells, where it occurs in synapse-like structures. Synapses are stable adhesive domains between two neighboring cells of multicellular organisms and function in cell-to-cell communication, as well as in information processing and storage. The synapse concept was developed more than 100 years ago for neuronal cell-to-cell communication, and it was recently adapted to other cell-to-cell communication mechanisms [42]. Successful cancer treatment targeted at the indecipherable intracellular interactomes is impossible. The development of efficient cancer therapies should focus on a new paradigm, advanced by the immune checkpoint therapy. Generally, this paradigm focuses on interactions between cancer and the stromal cells as therapeutic targets. It was suggested [30], that only direct interactions (for example, between ligands and their cognate receptors) form relatively simple binary contacts that are necessary for predictable therapeutic action. The synapse-like structures may universally mediate these interactions. An example is the successful immunotherapy of tumors based on the blocking of the immunological checkpoints (described in the paragraph below). We discuss details of the immunological synapses (ISs) as an example of the mechanism of stable intercellular interactions.

### 1.3. A Short Summary of the Formation of Immunological Synapses between T cells and the Activated Antigen-Presenting Cells

**Figure 1:**
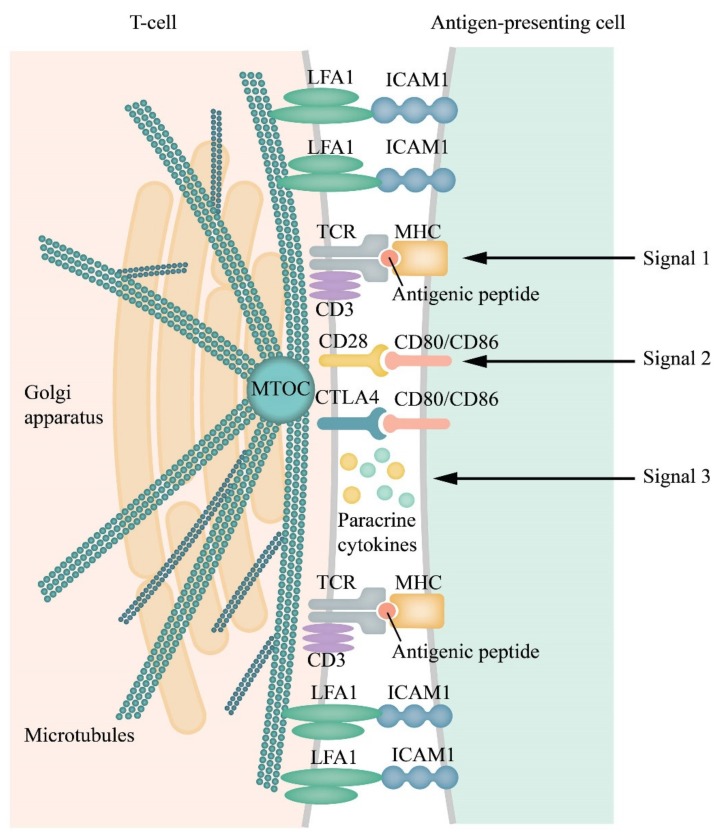
Scheme of an immunological synapse (IS) and receptor/(co-receptor)–ligand interactions within the synapse cleft and distribution of receptors and adhesion molecules in separate clusters within the IS. T cell receptor (TCR)/CD3 complex interacts with an MHC peptide. Adhesion molecules, such as lymphocyte function-associated antigen 1 (LFA-1) and Inter-Cellular Adhesion Molecule 1 (ICAM-1), on the surface of both cells, are responsible for the formation and stabilization of ISs, and initiation of signal pathways generated by the TCRs [47]. The cytoskeleton is remodeled, the Golgi apparatus, and the microtubule-organizing center (MTOC) move to the IS formation region [48,49]. All these rearrangements facilitate and allow the directed secretion within the synapse [49,50,51,52]. Activation/inhibition of T cells requires three signals. The first signal is initiated by binding of the TCR complexes with antigen peptides (blue circlet) presented by MHCs of the APCs. The second signal, an antigen-independent stage, is triggered by the interaction of the co-stimulating T cell receptor CD28 with ligands B7.1 (CD80) or B7.2 (CD86), delivered by the APCs (or tumor cells). Paracrine cytokines generate the third signal. All transmembrane contacts are clustered and have been symbolized by their pairs in the figure.

Activation of T cells requires three signals (Figure 1). The first signal is delivered through the interaction of T cell receptors (TCRs) with their antigen exposed on the surface of antigen-presenting cells (APCs) complexed with the proteins of the major histocompatibility complex (MHC). The second signal is antigen-independent and is delivered through the interaction between the stimulatory receptor C28 on the T cells and the protein CD80 (B7.1) or CD86 (B7.2) on the APCs [43]. The B7 family includes important membrane-bound ligands able to bind both co-stimulatory and co-inhibitory receptors (mentioned below). Stimulatory cytokines deliver the third signal in synapses. T cells, fully activated by all three signals, begin to proliferate and destroy carriers of antigens presented by the APCs. Apart from the co-stimulatory signals for T cells, there are also co-inhibitory signals produced by the T cells shortly after the initiation of the T cell proliferation [44,45,46]. Inhibitory interactions prevent overactive responses to the immune stimuli, thereby preventing autoimmune reactions.

The primary contact of TCRs with the antigen-loaded MHC proteins on the APCs (signal 1) induces the activation of multiple effectors including, among others, the membrane-bound integrins, the antigen-1 (LFA-1) associated with lymphocyte function and its ligand ICAM-1, signal adapters, and elements of the cytoskeleton and so on. These processes enhance interactions of the T cells with the APCs. Their contact also includes the co-stimulatory and co-inhibitory receptors (signal 2). These interactions culminate in the formation of ISs; narrow (12–15 nm) intercellular clefts where cytokines are concentrated, thereby enhancing the crosstalk between the cells (signal 3) [53]. ISs enable unique cell-to-cell interactions and are characterized by a number of essential features. These features include proximity of the immune cells and their target cells, strong intercellular adhesion, and secretion of signaling cytokines into the intercellular cleft [50,54,55,56]. However, data are showing that the T cells can function without forming synapses [53]. An essential characteristic of ISs is the mechanical forces generated due to intercellular adhesion. ISs that include the natural killer cells do not express TCRs but express activatory and inhibitory receptors that may regulate the transmission of signals and dynamic changes in the integrin-actin systems [57]. In general, the existing therapies targeted at blocking the co-inhibitory receptors affect the immunological synapses [50].

Here we have discussed the duration of IS existence and will not discuss the mechanism and kinetics of the IS formation, which have been discussed in recent reviews [55,58,59,60,61].

Cytotoxic lymphocytes (CTLs) form ISs, which only lasts a few minutes, owing to the death of target cells. This effect is probably due to the optimal CTLs function that may need fast and short-lived contact to kill as many target cells as possible. In contrast, the T lymphocytes form stable, long-lasting ISs (from 20–30 min to several hours), required for the directed and continuous secretion of cytokines [62]. These cytokines are located in secretory granules, and some of them undergo directed transport towards ISs. However, the transport of some cytokines, e.g., TNFs, is not directed, and the reasons for this difference remain unclear. 

### 1.4. Clusterization of Receptors and Ligands is A Prerequisite and Signature of IS Formation

An essential feature of ISs is the formation of receptor and ligand clusters, which mediate intercellular contacts. Some authors suggest the formation of synapse-like structures for all cases of membrane signalization. For example, it is indicated in Reference [63], “this in a way predicts a ‘synapse’ like entity for all membrane signaling events. Here there is no difference between a ligand/receptor pair induced higher-order lipid domain or one produced by a membrane curvature or any other biophysical means. The central purpose is to bring together enough sorted lipids and their associated protein receptors, and signaling ensues”.

**Figure 2:**
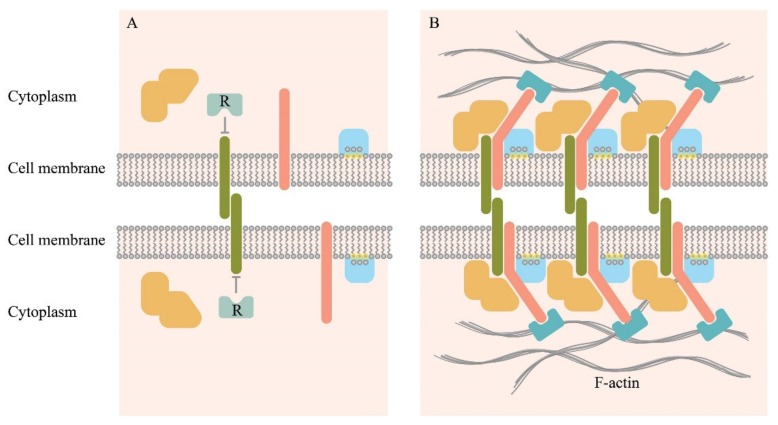
Schematic representation of individual molecules freely diffusing on the membrane surface (**A**), and a cluster of the intercellular adhesive complexes (**B**). Adhesion molecules (deep green) initiate binding, which also may involve other transmembrane proteins (pink), cytoplasmic proteins that can bind to the cytosolic part of the transmembrane proteins (orange). It also involves lipid groups present on the inner surface of the plasma membrane (yellow), and proteins with lipid-binding domains (light blue). Clustering may lead to the displacement of negative regulators associated with the cytosolic part of the adhesion molecules (R). Actin microfilaments stabilize macromolecular clusters through actin-binding proteins (cyan) [65].

In general, extracellular protein-protein interactions vary from very affine interactions with the equilibrium constant of dissociation (*Kd*) in the nanomolar to the picomolar range for soluble ligands. There are also extremely low-affinity interactions with the *Kd* within the micromolar to the millimolar range for the membrane receptor–ligand protein interactions [64]. Soluble ligands bind their receptors with high affinity because their concentration in the solution is usually low, and high-affinity binding ensures signal initiation. This effect is in contrast with the low affinity of the membrane-embedded proteins that often have a half-life of milliseconds in the monomeric state [64]. In this case, the strength of intercellular contacts depends on the clusterization of adhesion molecules comprising hundreds of receptors. This increases the avidity of the intercellular contact to a level sufficient to trigger a signaling event. Noteworthy, these adhesive events must be readily reversible. Clusterization and the associated transformations of the cytoskeleton have been shown schematically in Figure 2.

A relatively well-studied example is the clusterization of cadherins during the formation of the cadherin-mediated intercellular contacts [66]. The emergent intercellular adhesion is initiated by the binding of cadherin ectodomains on cell surfaces. Due to diffusion, the formed cadherin trans-dimers gather into small clusters at the sites of cell adhesion. With the participation of intracellular transformations of the cytoskeleton bound to the inner parts of the cadherins, the clusters are stabilized, and they expand. As a result, cell adhesion is enhanced strongly. Monomers and small inactive nanoclusters can coexist on the cell membrane. Small nanoclusters usually slowly diffuse or can be fixed through the actin cytoskeleton. The size of the nanoclusters in the ligand-free state may be probably below the functional threshold, and therefore, may be unable to stably bind their ligands and transmit a signal. On binding a ligand, the already existing small nanocluster can include accessory monomers.

Activation of the nanoclusters through binding ligands leads to an enlargement of nanoclusters, making them functional. Nanoclusterization is a general organization principle for many membrane receptors. It is rarely completed, and nanoclusters often coexist with randomly distributed non-clustered components. This coexistence may play a functional role or a regulatory role. Nanoclusters may function as complexes assembled in advance and capable of fast activation on binding a ligand [67]. A receptor cluster in the T cell synapses initiates the recruitment of hundreds of molecules to the membrane, interacts with the actin cytoskeleton¸ and plays a significant role in signal transmission. The formation of signal clusters leads to functional results that are difficult to predict from individual components [68]. This complex system interacts having emergent properties [69]. Transmission of intercellular adhesion signals in other cellular systems is similar to processes in the T cell immunological synapses. One of the recent examples is the ephrin type-A receptor 2 (EphA2)/EphrinA1 system that regulates cell adhesion, motility, and angiogenesis. The binding of EphA2 to EphrinA1 results in the formation of clusters that undergo actin-directed transport on the cell membrane [68]. These may display features similar to features found in a T cell immunological synapse. Clusterization provides stability for signaling by enhancing ligand-receptor functional local concentration and reducing the possible effect of the protein-degrading enzymes on the interaction result. Clusterization also results in higher specificity and provides an additional level of cell control [70,71]. 

A fundamental property of synapse is the proximity of the interacting cells. Such proximity was reported in an X-ray structural analysis of a CD200R and CD200 protein complex. CD200 (earlier known as OX2) is a widespread cellular surface protein that interacts with the receptor CD200R, expressed in the myeloid cells and some lymphoid cells. The authors calculated a distance of ~12 nm between the interacting cells, which corresponds to the spatial parameters of an immunological synapse. Since CD200 is also expressed in the non-lymphoid cells, synapse-like interactions may be widely used [72,73].

In summary, one of the essential features of the synapse-like intercellular contacts is the presence of receptor clusters on one of the interacting cells and ligand clusters on the other. These clusters are associated with the remodeling of the intracellular cytoskeletons. This allows the polarization of the cell secretory mechanism in immunological synapses, which provides another feature of synapse-directed secretion [49]. The existence of such membrane ligand-receptor pair clusters on the interacting cells should imply the existence of synapse-like structures [63,72,74]. 

### 1.5. Remodeling of Cytoskeletons in Intercellular Interactions

Intercellular interactions induce a radical remodeling of the cytoskeleton (Figure 2). As a result, the Golgi apparatus moves to the IS, thereby allowing directed secretion within the synapse (Figure 1). The location of the centrosome is also drastically changed upon recognition of the target cell. The centrosome moves from the back-end of the cell to its front edge where a synapse forms [48,49,50,51,52]. The involvement of the cytoskeleton in cluster formation has been shown schematically in Figure 2. This process is rather well-studied for the E-cadherin-mediated intercellular interactions. It involves the p120 catenin that, together with the beta- and the alfa-catenins, binds the cytoplasmic domain of cadherin. Alfa-catenin directly binds F-actin. This process stabilizes the clusterization of cadherin [49,66,75].

Adhesion induces remodeling of the cytoskeleton and affects the cell polarity, as discussed above. It is also related to some cellular processes, including differentiation and proliferation. Disorders of cell polarity are associated with disorders of development. Therefore, many tumors show the loss of E-cadherin-mediated intercellular adhesion [76]. These complex processes have a genetic basis and an epigenetic basis that is mostly unclear. In recent years, there have been attempts to decipher it, and some representative results have been presented below. An extensive siRNA screening revealed tens of genes that were probably involved in the regulation of adhesion (see the review [77]), through involvement in β-catenin and β1-integrin pathways, regulation of the actin cytoskeleton, and EGFR signaling. Noteworthy, among the genes mutated in lung carcinoma, a significant proportion of genes participate in the regulation of the cytoskeleton state, including the genes *IQGAP3*, *EPB41*, *CDC42*, *PARD6G*, *PTK2B*, and *KALRN*. The proportion of these genes has been found to increase in metastases. This suggested the involvement of these genes in the process of metastasis [78]. The transcription factor GATA4, crucially important in the early liver development, has been shown to be involved in the pathophysiology of hepatoblastoma, an embryonic tumor of childhood. Suppression of the *GATA4* gene (using RNA interference) disturbed the migration of the human hepatoblastoma cells, HUH6. Moreover, the expression of genes involved in the cytoskeleton organization, intercellular adhesion, and dynamics of the extracellular matrix was found to be changed. One hundred and six differentially expressed genes (34 up-regulated and 72 down-regulated) were identified [79].

Furthermore, the relationships between several proteins involved in intercellular adhesion have been identified, and relentless efforts continue to determine the full range of such proteins, especially those regulating these processes [80]. In particular, 27 genes have been identified in which mutations disrupt intercellular adhesion during collective migration. For example, p73, which is important for folliculogenesis of the ovaries, functions as a vital regulator of a gene network involved in cell-to-cell adhesion and migration [81]. The nuclear retinoic acid receptors (retinoic acid receptor alpha, RARα, retinoic acid receptor beta, RARβ, and retinoic acid receptor gamma, RARγ) are ligand-dependent transcription factors regulating the expression of genes related to cell differentiation and proliferation. A whole-genome analysis has been performed for the RAR-regulated genes in the mouse embryonic fibroblasts (MEFs) with a comparison of the wild type MEFs with MEFs having all three RARs knocked out [82]. The absence of RARs was found to be associated with cell adhesion, and the knock-out MEFs were unable to adhere and to spread on substrates and displayed a disrupted network of actin filaments.

Although a relationship between metabolism and cell adhesion has been reported, the exact molecular details of their interaction remain to be understood. Minsky et al. [83] showed that PGC-1α, a major transcription co-activator of metabolic gene expression, takes part in inhibiting the expression of cell adhesion genes. Using cell lines, primary cells, and mice, the authors demonstrated that both endogenous and exogenous PGC-1α inhibited the expression of different cell adhesion molecules. In addition, PGC-1α modulates the adhesion of primary fibroblasts and the hepatic stellate cells to the proteins of the extracellular matrix. These results outlined the relationship between central pathways controlling metabolic regulation and cell adhesion and identified PGC-1α as one of the connecting links between these major cell networks [83]. The examples mentioned above show that although the problem is of fundamental interest, the data available now are too scarce. They do not allow deduction of the molecular genetic mechanisms of remodeling cytoskeletons and the formation of the ligand-receptor clusters in the process of cell-cell adhesion.

### 1.6. Circulating Cancer Cells Form Clusters through Tomo- and Heterotypic Intercellular Adhesions That Are Responsible for Metastasis and Possess the Stemness Property

Carcinoma cells can metastasize, still maintaining cell–cell contacts [84,85,86]. One reason for this may be that the epithelial cancer cells use stromal cells during invasion [87,88] (see more detail below). Solid tumors secrete a large number of highly heterogeneous circulating tumor cells (CTCs) into the bloodstream [89,90,91,92,93,94,95]. Still, only a small proportion of the CTCs (0.2% reported by Tripathi et al. [96]) can survive and ultimately result in metastatic changes. Efficient metastasis (>90% [96]) has been attributed to the CTCs clusters, sometimes referred to as the circulating tumor microemboli [94], defined as groups of two or more aggregated CTCs. According to the estimates, tumor cells detach from the primary tumor at 3.2 × 10^6^ cells per gram of tumor per day, but more than half of the detached tumor cells die. Approximately only one cell per 10^6^–10^7^ leukocytes remain [94]. The molecular mechanisms responsible for the formation and spread of clusters, and the pathways supporting their survival and metastatic potential remain mostly unknown [93]. 

Most data on CTC clusters participation in metastasis describe homotypic clusters [97,98]. It is evident that adhesion and cytoskeleton processes actively participate in such kind of clusterization. Furthermore, changes in the cell adhesion properties are required to establish and maintain the trait of cancer cell stemness [99]. Persistent and adhesion-dependent survival signals in the CTC clusters can support the survival stimuli, thereby facilitating active metastases. While individual CTCs may experience problems with survival, such as oxidative stresses and immune effects, leading to apoptosis, the CTCs in clusters remain protected [92]. In particular, the CD44-dependent aggregation in blood circulation confers traits to the CTC clusters that are similar to those of cancer stem cells, which leads to a more efficient metastasis in the secondary organs [97,98]. 

However, CTCs can also contain other components, such as leukocytes, endothelial cells, platelets, and cancer-associated fibroblasts (CAFs) that provide a microenvironment favorable for survival [93]. The role of CAFs in metastasis has been widely studied [100,101,102]. The interactions between CAFs and cancer cells were reported to produce a reciprocal and convergent set of signaling activities that promote cancer invasion and metastasis [24]. Santi et al. suggested that cancer and stromal cells of invasive tumors may have been in direct contact and may have established complex crosstalk during tumor development [98]. CAFs induce the formation of metastasizing clusters of tumor cells, with the participation of an intercellular adhesion [103]. According to the authors, CAFs may drive the formation of tumor cell clusters composed of two distinct cancer cell populations, one in a highly epithelial state and another in a hybrid epithelial/mesenchymal state and confer invasive and metastatic traits upon tumor cells. The stromal cell-derived factor 1 (SDF-1) and transforming growth factor-β (TGF-β) mediate the tumor cell cluster formation, invasion, and metastasis via Src activation. The authors also detected in cancer cells, CAFs induced cell–cell adhesion molecules (E-cad, CAM5, or CAM), causing the formation of tumor cell clusters. One can suggest that these same molecules take part in the adhesion between CAFs and tumor cells, providing a tight contact (synapse?) for efficient SDF-1 and TGF-β crosstalk. Following the above data, CAF, as has been shown [104], can promote aggressive metastatic phenotypes of non-invasive bladder cancer cells through an EMT induced by the secretion of IL-6. A critical study [105] showed that CAFs induced invasion through a heterophilic adhesion to both the participating N-cadherin on the membranes of CAFs and the E cadherin on the membranes of the cancer cells. The weakening of this adhesion blocked the ability of the CAFs to direct the collective migration of cells and cancer cell invasion. Nectins and afadin (organizers of cell contacts) were recruited simultaneously to interfaces between the CAFs and the cancer cells. These data suggest that active heterophilic adhesion between CAFs and cancer cells may lead to a cooperative tumor invasion. Contacts between the CAFs and the cancer cells may be formed due to interactions of the Eph-receptors and the corresponding ephrine ligands [106]. It suggests that these direct contacts may form synapse-like structures that may enhance the paracrine communications. One of these communication strategies may be the directed secretion of soluble growth factors and chemokines [105].

A remarkable example of direct contacts between the stromal (the fibroblasts and the mesothelial cells) and the cancer cells can be seen in spheroids of the ovarian carcinoma ascites [107,108,109,110]. When in the abdominal cavity, tumor cells combine with the free-floating myofibroblast cells forming multicellular heterotypic spheroids. This enables the tumor cells to avoid anoikis and acquire a more invasive phenotype. Macrophages have also been demonstrated to play an active role in the formation of spheroids [111]. The multicellular spheroids attach to the mesothelial cells using various cell adhesion molecules. Adhesion molecules, including integrins and cadherins, mediate adhesion between cells and cell interaction with the extracellular matrix and play a role in the formation and metastasis of ovarian cancer [112]. However, the mechanisms of CAFs–cancer cell interactions during collective migration are still far from being investigated. In particular, the question of whether the signaling clusters are formed between the two entities remains untouched.

### 1.7. Why are CAFs “Chosen” for Cancer Cell Partners and Direct Contacts

Cancer-associated fibroblasts (CAFs) are ideal stromal partners for the collective invasion of cancer cells [87,113]. The CAFs were shown to be one of the predominant cell types in the stroma [21,23,24,27,29,113]. They are a heterogeneous cell “family” or a “group” demonstrating mesenchymal-like properties. CAFs are often close to or in direct contact with the tumor cells [23,24,27,114]. However, only a few studies have provided experimental data supporting the direct interaction of CAFs and cancer cells and its functional consequences. It has been hypothesized that the transformation of normal fibroblasts into CAFs occurs due to the continuous signals from the malignant cells [115,116,117,118]. In response, CAF populations produce paracrine signals, which affect cancer progression. The most evident and important consequence of such an interaction is the involvement of CAFs in the stimulation of EMT of cancer cells, as well as in their invasion and metastasis [87,100,105,119,120,121,122], as a special case of collective cell migration typical for multicellular organisms [123]. Gaggioli et al. [87] showed that in collectively invading co-cultures of the squamous cell carcinoma (SCC) cells and the stromal fibroblasts, the leading cells were always the fibroblasts. The special tests demonstrated that the invasion by the SCC cells requires either proximity to or direct contact with the CAFs. One more argument for this can be found in the review by Yamaguchi et al. [122].

To study the input of the direct intercellular contacts and the paracrine signal factors in the metastasis of the non-small cell lung carcinoma (NSCLC) cells, Choe et al. [120] used two variants of co-culturing. These included direct co-culturing of an NSCLC cell line with the primary CAF cultures from patients with a resected NSCLC, and an indirect co-culturing with a permeable membrane. In these experiments, the CAFs induced an EMT more actively in direct co-culturing, indicating that physical contacts between the NSCLC cells and the CAFs can control the metastatic potential of the NSCLC cells. It does not exclude the possible role of the paracrine interaction. This is enhanced by the physical cell interactions similar to that in immunological synapses. A review by Santi et al. [100] contains data showing that the CAFs adjacent to the cancerous regions can increase the invasiveness of the cancer cells under cell–cell interactions assisted by various pro-invasive molecules, such as cytokines, chemokines, and inflammatory mediators.

The malicious role of the direct CAF contacts with the cancer cells makes the disruption of these contacts an important target for cancer therapy. Yamaguchi et al. [122] attempted to find the inhibitors of direct interactions between the CAFs and the cancer cells. They found that the Src inhibitor, Dasatinib, efficiently blocked the physical bonds between the CAFs and the scirrhous gastric cancer (SGC) cells with a minimal cytotoxic effect. Dasatinib was also effective against the peritoneal dissemination of SGC cells in a mouse model. According to histological analysis, mice treated with Dasatinib were found to contain fewer metastasizing tumors associated with the stromal fibroblasts than in the controls. It implies that the direct interaction between the CAFs and the SGC cells can be a target for anti-metastasis therapy [122]. However, the authors recommended that the results be treated with caution as a decrease in CAF levels led to a faster progression of pancreatic cancer. Despite the inconsistency of these results, they emphasized the requirement for safety tests for inhibitors of the CAF–cancer cell interactions in anti-cancer therapy. In contrast, using the CAF–cancer contacts instead of the CAFs as therapeutic targets is a safer approach, as this strategy will not affect the CAF levels.

## 2. Conclusions

### The Power of Clusters in Signal Transmission, and Their Vulnerability to a Directed Disruption

Any intercellular recognition between two membranes may include hundreds, possibly thousands, of receptors that may enhance the avidity of intercellular contacts to a level sufficient to trigger a signal event [63,64,124].

Synapse-like structures can be identified by several features:

1. The proximity of the interacting cells.
2. The presence of receptor clusters and corresponding ligands on the interacting cells.
3. The presence of strong interactions that allow cancer cells to migrate together with the stromal cells within circulating clusters.
4. A remodeled cytoskeleton in the interacting cells.
5. Characteristic changes in the transcription regulation [81] and possible epigenetic changes.

The detection of synapse-like structures that emerge during the interaction of cancer and the stromal cells, mostly with the CAFs, will open a new dimension in cancer treatment. This may supplement the immune checkpoint therapy, which is also targeted at disrupting synapses between the cancer cells and cells of the immune system. The formation of the clusters suggests that several different incoming signals could already be integrated at the plasma membrane level via direct allosteric interactions between the protomers that form the cluster [125]. It should lead to the emergence of new unpredictable features different from those expected from the properties of the interacting monomeric ligand-receptor pairs. The elucidation of these properties can open new therapeutic horizons. The proximity of adhesion molecules in clusters in itself opens up new possibilities for therapeutic agents directed at nearby receptor-ligand pairs in the clusters. For example, the application of bivalent ligands composed of two functional pharmacophores linked by a spacer. This is considered in pharmacology as one of the most promising strategies for the treatment of homo or heterodimeric receptors (see, for example, [125,126]. Such kind of therapy may be a new way of tumor destruction.

The above pertains to cancerous tumors and their metastasis, and there is no doubt that these processes involve many, if not all, cells of the stromal environment of cancer. Studies will be needed on the selection of the most “malicious” partners of the cancer cells that protect them from a therapeutic action and facilitate their proliferation and metastasis. Among these partners are CAFs that, as suggested in this review, may interact with the cancer cells forming synapse-like structures. It justifies the title of a paper [127]: “Cancer-associated-fibroblasts and tumor cells: a diabolic liaison driving cancer progression”. Disrupting these detrimental connections is a challenging but still achievable and promising task.
